# Chronische *Tropheryma-whipplei*-Infektion: Eine wichtige Differentialdiagnose der therapierefraktären Polyarthritis

**DOI:** 10.1007/s00393-022-01194-5

**Published:** 2022-04-06

**Authors:** Nikolas Ruffer, Marie-Therese Holzer, Yannik Gkanatsas, Izabela Schinglerová, Damir Boro, Martin Krusche, Ina Kötter

**Affiliations:** 1 Klinik für Rheumatologie und Immunologie, Klinikum Bad Bramstedt, Bad Bramstedt, Deutschland; 2 III. Medizinische Klinik, Sektion Rheumatologie, Universitätsklinikum Hamburg-Eppendorf, Martinistr. 52, 20246 Hamburg, Deutschland

**Keywords:** Morbus Whipple, Gelenkinfektion, Seronegative rheumatoide Arthritis, Psoriasisarthritis, Gelenkpunktat, Whipple’s disease, Joint infection, Seronegative rheumatoid arthritis, Psoriatic arthritis, Synovial fluid

## Abstract

**Hintergrund:**

Therapierefraktäre Arthritiden sind ein häufiges Problem im rheumatologischen Alltag und können eine differentialdiagnostische Herausforderung darstellen. Chronische Infektionen durch *Tropheryma whipplei *(*T. whipplei*) sollten in diesen Fällen bedacht werden.

**Ziel der Arbeit:**

Anhand von 5 klinischen Fällen werden in dieser fallbasierten Übersichtsarbeit die diagnostischen und therapeutischen Prinzipien im Management der chronischen *T.-whipplei*-Infektion erläutert.

**Ergebnis:**

Der Morbus Whipple ist eine infektiöse Multisystemerkrankung, die durch das Bakterium *T. whipplei* ausgelöst wird. Typischerweise manifestiert sich die Erkrankung mit Arthralgien, Gewichtsverlust und Diarrhoen. Die Gelenkmanifestationen gehen den gastrointestinalen Krankheitserscheinungen häufig mehrere Jahre voraus. Neben systemischen Manifestationen (Morbus Whipple) kann *T. whipplei* auch zu lokalisierten Infektionen der Gelenke ohne gastrointestinale Beteiligung führen. Die Gelenkmanifestationen systemischer und lokalisierter *T.-whipplei*-Infektionen werden fälschlicherweise häufig als Zeichen verschiedener autoimmunologischer Arthritiden gedeutet.

**Diskussion:**

Bei der Abklärung therapierefraktärer Arthritiden sollte an einen Morbus Whipple und lokalisierte Gelenkinfektionen durch *T. whipplei* gedacht werden. Diagnostisch wegweisend ist die Untersuchung des Gelenkpunktates auf *T. whipplei* mittels Polymerasekettenreaktion.

## Abstract (en)

**Background:**

Refractory arthritis is a common problem in routine rheumatology practice, and can be a diagnostic challenge. In these cases, chronic *Tropheryma whipplei* (*T. whipplei*) infection is an important differential diagnosis that should be considered.

**Objective:**

Based on five clinical cases, this case-based review describes the diagnostic and therapeutic principles in the management of chronic *T. whipplei* infection.

**Results:**

Whipple’s disease is a multisystemic infectious disease caused by the bacterium *T. whipplei*. The disease typically manifests with arthralgia, weight loss and diarrhoea. Joint involvement often develops years before gastrointestinal symptoms occur. In addition to systemic manifestations (“classic Whipple’s disease”), *T. whipplei* can also lead to localized joint infections without gastrointestinal involvement. Articular manifestations of systemic and localized *T. whipplei* infections are commonly misdiagnosed as a sign of various forms of autoimmmune arthritis.

**Discussion:**

Whippleʼs disease and localized *T. whipplei* joint infection should be considered in the diagnostic work-up of refractory arthritis. Synovial fluid analysis by means of specific polymerase chain reaction-based testing for *T. whipplei* is diagnostically ground-breaking.

## Einleitung

Therapierefraktäre Arthritiden stellen eine Herausforderung im rheumatologischen Alltag dar, welche Anlass zu vielfältigen differentialdiagnostischen Überlegungen gibt. In diesen Fällen sollte eine kritische Reevaluation der initialen Diagnose einer entzündlich-rheumatischen Grunderkrankung (z. B. seronegative rheumatoide Arthritis) erfolgen. Insbesondere Infektionen mit „atypischen“ Erregern können häufig rheumatologische Krankheitsbilder imitieren („mimics“). In diesem Zusammenhang ist die chronische Infektion mit *Tropheryma whipplei *(*T. whipplei*) eine wichtige Differentialdiagnose, da diese häufig erst spät im Krankheitsverlauf erkannt wird. Die Gelenkmanifestationen beim Morbus Whipple gehen der gastrointestinalen Symptomatik in fast drei Viertel der Fälle um 6 bis 7 Jahre voraus [1] und erschweren daher die Unterscheidung von Arthritiden autoimmuner Genese.

Anhand von 5 klinischen Fällen werden in dieser fallbasierten Übersichtsarbeit die diagnostischen und therapeutischen Prinzipien im Management der chronischen *T.-whipplei*-Infektion erläutert sowie „red flags“ als Hinweis auf eine chronische *T.-whipplei*-Infektion beschrieben.

## Fallberichte

### Fall 1

Ein 50-jähriger Patient mit vordiagnostizierter „Psoriasisarthritis (PsA) sine Psoriasis“ wurde mit rezidivierenden Polyarthritiden und immobilisierenden Schmerzen der Lendenwirbelsäule stationär aufgenommen. In der Vorgeschichte fanden sich erfolglose Therapieversuche mit verschiedenen Basistherapeutika (disease-modifying anti-rheumatic drugs, DMARDs).

In der klinischen Untersuchung zeigte sich eine Tenosynovialitis im Handgelenk links sowie ein Erguss im Sprunggelenk links und Ellenbogengelenk rechts. Das C‑reaktive Protein (CRP) war leichtgradig erhöht (2,1 mg/dl; < 0,5 mg/dl). Die Autoimmunserologie war unauffällig. Eine Gelenkpunktion des rechten Ellenbogengelenkes ergab den Befund einer Arthritis mit Nachweis von *T. whipplei* (400.000 Genomäquivalente/ml) mittels Polymerasekettenreaktion (PCR). Eine Ösophagogastroduodenoskopie (ÖGD) mit Duodenalbiopsien ergab sowohl histologisch als auch molekularbiologisch (PCR) keine weiteren Hinweise für einen Morbus Whipple.

20 Monate nach Einleitung einer antibiotischen Therapie mit Ceftriaxon 2 g i.v. täglich über 14 Tage und anschließender Therapie mit Cotrimoxazol 960 mg p.o. 2 × täglich für weitere 12 Monate ist der Patient bei normwertigen Entzündungsparametern anhaltend beschwerdefrei.

### Fall 2

Ein 54-jähriger Patient mit vordiagnostizierter „Spondyloarthritis (SpA) mit peripherer Gelenkbeteiligung DD seronegative rheumatoide Arthritis (RA)“ wurde zur Abklärung einer therapierefraktären Polyarthritis stationär aufgenommen. Der Patient profitierte nicht von einer systemischen Glukokortikoidtherapie. Neben den Gelenkbeschwerden berichtete der Patient unter rezidivierenden Bauchschmerzen und Obstipation zu leiden.

Laborchemisch fanden sich erhöhte Entzündungsparameter (CRP 4,0 mg/dl). Rheumafaktor (RF) oder Antikörper gegen zyklische citrullinierte Peptide (Anti-CCP-Ak) ließen sich nicht nachweisen. Klinisch und sonographisch zeigte sich das Bild einer Arthritis der Sprunggelenke beidseits. Zusätzlich konnte sonographisch eine Arthritis der Calcaneocuboidalgelenke beidseits mit ausgeprägter Synovialishypertrophie und Gelenkergüssen nachgewiesen werden. Die PCR aus dem Gelenkpunktat des rechten Sprunggelenkes war positiv für *T. whipplei* (20.000 Genomäquivalente/ml). Eine ÖGD und Liquorpunktion ergaben histologisch bzw. molekularbiologisch keine weiteren Hinweise für einen Morbus Whipple.

12 Monate nach Einleitung einer antibiotischen Therapie mit Ceftriaxon 2 g i.v. täglich über 14 Tage und anschließender Therapie mit Cotrimoxazol 960 mg p.o. 2 × täglich ist der Patient anhaltend beschwerdefrei. Parallel hierzu kam es zu einer Regredienz des CRP (1 mg/dl). Die Cotrimoxazoltherapie konnte daher nach einem Jahr beendet werden.

### Fall 3

Eine 72-jährige Patientin mit vordiagnostizierter „PsA DD seropositive RA“ wurde aufgrund einer therapierefraktären Polyarthritis stationär aufgenommen. Zu Beginn der Erkrankung bestand ein Ansprechen auf Prednisolon, Methotrexat und Etanercept. Andere Basistherapeutika hatten im Verlauf keinen anhaltenden Therapieerfolg erzielt.

Klinisch und sonographisch ergab sich der Befund einer aktiven Polyarthritis mit synovialer Proliferation im Bereich der Finger‑, Hand‑, Knie‑, Ellenbogen- und Schultergelenke beidseits. Laborchemisch bestand eine Entzündungskonstellation (CRP 4,11 mg/dl) sowie eine hypochrome, normozytäre Anämie (Hämoglobin [Hb] 9,6 g/dl) mit Eisen- und Vitamin-B6-Mangel. Die Autoimmunserologie war schwach positiv für RF (6,7 U/l; Referenz: < 5,0 U/l).

**Figure 1:**
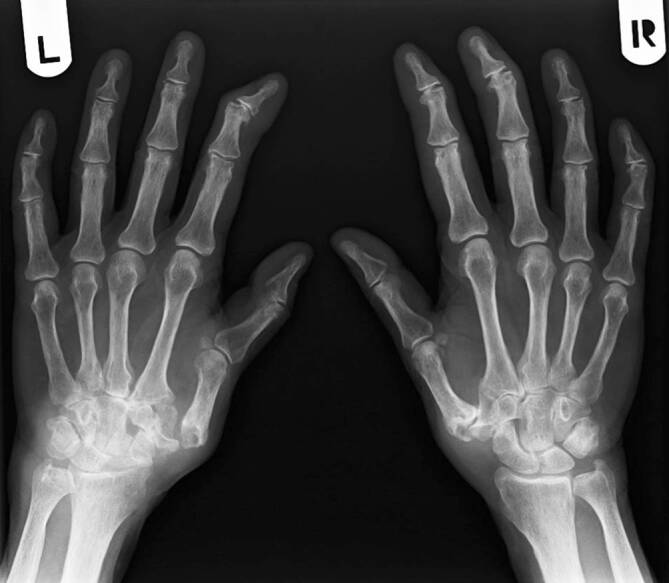

Nativradiologisch war im Bereich der Hände ein Mischbild aus erosiver Polyarthritis (Stadium IV nach Larsen) sowie Heberden- und Bouchard-Arthrosen beidseits sichtbar (Abb. 1). Zusätzlich fanden sich mutilierende Veränderungen der Handgelenke beidseits. Eine Magnetresonanztomographie der Halswirbelsäule zeigte eine aktive Arthritis im Kiefergelenk rechts und im Atlantodentalgelenk.

Die Untersuchung des Gelenkpunktates aus dem linken Kniegelenk zeigte einen entzündlichen Erguss mit positiver PCR für *T. whipplei* (400.000 Genomäquivalente/ml). Die histopathologische und molekularbiologische Untersuchung von makroskopisch milchig-weißlichen Schleimhautveränderungen im Duodenum ergab hingegen keinen Hinweis für einen Morbus Whipple. Eine Lumbalpunktion wurde von der Patientin abgelehnt. Unter einer antibiotischen Therapie mit Ceftriaxon 2 g i.v. täglich über 14 Tage und anschließender Therapie mit Cotrimoxazol 960 mg p.o. 2 × täglich waren die Entzündungsparameter und Anämie rückläufig. Nachdem die Therapie mit Cotrimoxazol vorzeitig nach 3 Monaten durch die Patientin beendet wurde, entwickelte sich im Verlauf ein Rezidiv der Polyarthritis. In einer erneuten Gelenkpunktion ließ sich *T. whipplei* jedoch nicht nachweisen, sodass wir von einer komorbiden immunologischen Arthritis ausgingen. Da nach erneuter Einleitung von Cotrimoxazol in Kombination mit Hydroxychloroquin nur eine partielle Besserung der arthritischen Beschwerden zu erzielen war, erfolgte ergänzend ein Prednisolonstoß. Bei gutem Glukokortikoidansprechen der Gelenke, jedoch persistierender Psoriasis entschied man sich aufgrund des günstigen Sicherheitsprofils für die additive Gabe von Guselkumab.

### Fall 4

Ein 75-jähriger Patient mit vordiagnostizierter „seronegativer RA“ wurde aufgrund therapierefraktärer rezidivierender Polyarthritiden, Gewichtsverlust und Inflammation unklarer Ursache stationär aufgenommen. Eine externe Computertomographie (CT) des Abdomens hatte eine mesenteriale Lymphadenopathie gezeigt. Eine kurz vor Aufnahme durchgeführte ÖGD mit Duodenalbiopsien hatte histologisch keinen Hinweis für einen Morbus Whipple ergeben.

Klinisch bestand eine ausgeprägte Kachexie (10 kg Gewichtsverlust in 9 Monaten). Laborchemisch ergab sich eine entzündliche Laborkonstellation (CRP 6,22 mg/dl) mit Leukozytose und normochromer, normozytärer Anämie (Hb 10,8 g/dl). RF oder Anti-CCP-Ak ließen sich nicht nachweisen. Sonographisch zeigten sich Gelenkergüsse der Kniegelenke beidseits und des linken Sprunggelenkes mit geringer Synovialitis sowie eine Splenomegalie (146 × 110 × 51 mm). Die PCR aus den Gelenkpunktaten des Kniegelenkes rechts (450.000 Genomäquivalente/ml) und Sprunggelenkes links (400.000 Genomäquivalente/ml) war positiv für *T. whipplei*. Eine PCR-Untersuchung der initial entnommenen Duodenalbiopsien war ebenfalls positiv für *T. whipplei*. Die Untersuchung des Liquors war unauffällig.

Etwa 4 Stunden nach Beginn einer antibiotischen Therapie mit Ceftriaxon 2 g i.v. entwickelte der Patient Fieber und hypertensive Blutdruckwerte, was wir als Jarisch-Herxheimer-Reaktion werteten. Die Reaktion bildete sich innerhalb von Stunden spontan zurück. Innerhalb von wenigen Tagen kam es zu einer Normalisierung der zuvor chronisch erhöhten Entzündungsparameter. Nach 6 Monaten antibiotischer Therapie mit Cotrimoxazol 960 mg p.o. 2 × täglich bildeten sich auch die Blutbildveränderungen und Splenomegalie zurück. Eine Fortführung der antibiotischen Therapie für weitere 6 Monate ist angestrebt.

### Fall 5

Eine 71-jährige Patientin mit vordiagnostizierter „seronegativer RA DD PsA“ wurde aufgrund einer akuten Arthritis des linken Kniegelenkes und rechten Sprunggelenkes stationär aufgenommen. Rezidivierende Arthritiden bestanden seit Jahren mit wechselnder Lokalisation. In der Vorgeschichte war ein paramedianer Ponsinfarkt rechts mit spastischer Hemiparese links bekannt, die sich in den Tagen vor der stationären Übernahme verschlechtert hatte.

Laborchemisch zeigte sich eine deutliche Entzündungskonstellation (CRP 13,76 mg/dl). Die PCR aus den Gelenkpunktaten des Kniegelenkes links (900.000 Genomäquivalente/ml) und Sprunggelenkes rechts (500.000 Genomäquivalente/ml) sowie eine anschließend durchgeführte Liquoruntersuchung (1000 Genomäquivalente/ml) waren positiv für *T. whipplei*. Die histopathologische Untersuchung von Duodenalbiopsaten ergab hingegen keinen Hinweis für einen Morbus Whipple. Allerdings konnte die PCR genetisches Material von *T. whipplei* nachweisen.

Nach Einleitung einer antibiotischen Therapie mit Ceftriaxon 2 g i.v. täglich über 14 Tage und anschließender Therapie mit Cotrimoxazol 960 mg p.o. 2 × täglich zeigte sich innerhalb von 2 bis 3 Wochen eine Besserung der Hemispastik und Gelenkmanifestationen sowie eine Normalisierung der Entzündungsparameter. 6 Monate nach Therapiebeginn sind die Entzündungsparameter weiterhin normwertig und die Patientin ist bezüglich der arthritischen Symptomatik beschwerdefrei. Die Fortführung der Therapie ist für weitere 6 Monate geplant.

## Ergebnisse

Das Leitsymptom der beschriebenen Fälle ist die rezidivierende Polyarthritis ohne Ansprechen auf immunsuppressive Therapien. Letztendlich konnte in allen Fällen die Diagnose einer chronischen Infektion durch *T. whipplei* gestellt werden. Diagnostisch wegweisend war die PCR-Untersuchung des Gelenkpunktates auf *T. whipplei*. Interessanterweise zeigte nur ein Patient typische gastrointestinale Symptome eines Morbus Whipple (Fall 4: Gewichtsverlust, Kachexie) mit gleichzeitigem molekularbiologischem Nachweis von *T. whipplei* in den Duodenalbiopsaten. Die klassischen histologischen Befunde eines Morbus Whipple (PAS^1^-positive Einschlüsse in Makrophagen der duodenalen Lamina propria) wurden bei keinem Fall in den Duodenalbiopsaten nachgewiesen. Bei einer Patientin (Fall 5) ließ sich eine zentralnervöse Infektion durch die PCR-Untersuchung des Liquors bestätigen.

Eine Übersicht der geschilderten Fälle gibt Tab. 1.

**Table 1:** 

Fälle	Alter	Geschlecht	Vordiagnose	Vortherapien	Dauer bis Diagnose (Jahre)	Klinik	Labor	T.-whipplei-Diagnostik
Fall 1	50	Männlich	PsA	GK, LEF, APR, GOL, MTX, ADA	9 Jahre	Polyarthritis	RF −	Synovia-PCR +
						LWS-Schmerzen (MRT mit KM: KM-Enhancement der kleinen Wirbelgelenke LWK5/SWK1, Enthesitis der Ligamenta interspinalia und supraspinalia)	Anti-CCP −	ÖGD-Histo −
							CRP 2,1 mg/dl	ÖGD-PCR −
							Hb 13,3 g/dl	LP-PCR ø
Fall 2	54	Männlich	SpA, RA, ReA	GK, MTX, LEF, ADA, ETC, CER, TCZ, HCQ	9 Jahre	Polyarthritis	RF −	Synovia-PCR +
						Bauchschmerzen	Anti-CCP −	ÖGD-Histo −
						Obstipation	CRP 4,0 mg/dl	ÖGD-PCR −
							Hb 14,1 g/dl	LP-PCR −
Fall 3	72	Weiblich	PsA, RA	GK, MTX, RTX, ADA, ABA, IXE, ETC, CER, APR, SEC, UST, UPA	30 Jahre	Erosive Polyarthritis	RF (+)	Synovia-PCR +
						Arthritis des Atlantodentalgelenkes und Kiefergelenkes rechts (MRT-HWS)	Anti-CCP −	ÖGD-Histo −
						HWS-Instabilität	CRP 4,1 mg/dl	ÖGD-PCR −
						Kribbelparästhesien (Hinterkopf, Extremitäten)	Hb 9,6 g/dl	LP-PCR ø
						Anämie		
Fall 4	75	Männlich	RA	GK, MTX, ADA, TCZ	1 Jahr	Polyarthritis	RF −	Synovia-PCR +
						Bursitis trochanterica	Anti-CCP −	ÖGD-Histo −
						Gewichtsverlust, Kachexie	CRP 6,2 mg/dl	ÖGD-PCR +
						Nachtschweiß	Hb 10,8 g/dl	LP-PCR −
						Splenomegalie		
						Mesenteriale Lymphadenopathie		
						Anämie		
Fall 5	71	Weiblich	RA, SpA, PsA	GK, MTX, SUL	22 Jahre	Polyarthritis	RF −	Synovia-PCR +
						Nachtschweiß	Anti-CCP −	ÖGD-Histo −
						Progrediente Hemispastik links (Z. n. Ponsinfarkt)	CRP 13,7 mg/dl	ÖGD-PCR +
							Hb 13,6 g/dl	LP-PCR +

*ABA* Abatacept; *ADA* Adalimumab; *APR* Apremilast; *Anti-CCP* Antikörper gegen zyklische, citrullinierte Peptide; *CER* Certolizumab; *CRP* C-reaktives Protein (< 0,5 mg/dl); *ETC* Etanercept; *GK* Glukokortikoide; *GOL* Golimumab; *HCQ* Hydroxychloroquin; *Hb* Hämoglobin (Frauen 12–16 g/dl, Männer 14–18 g/dl); *Histo* histologische Untersuchung; *HWS* Halswirbelsäule; *IXE* Ixekizumab; *KM* Kontrastmittel; *LEF* Leflunomid; *LP* Lumbalpunktion; *LWS* Lendenwirbelsäule; *MRT* Magnetresonanztomographie; *MTX* Methotrexat; *ÖGD* Ösophagogastroduodenoskopie; *PCR* Polymerasekettenreaktion; *RF* Rheumafaktor; *RTX* Rituximab; *SEC* Secukinumab; *SUL* Sulfasalazin; *TCZ* Tocilizumab; *UPA* Upadacitinib; *UST* Ustekinumab

*+* positiv; *(+)* schwach positiv; *−* negativ; *ø* nicht durchgeführt

## Diskussion

### Chronische *T.-whipplei*-Infektion: Morbus Whipple und lokalisierte Infektionen

Klinisch können verschiedene Manifestationen einer *T.-whipplei*-Infektion unterschieden werden [2, 3]: (1) Morbus Whipple („classic Whipple’s disease“), (2) lokalisierte Infektionen (z. B. Endokarditis, isolierte Arthritis), (3) akute Infektionen und (4) asymptomatische Träger.

Der Morbus Whipple ist eine infektiöse Multisystemerkrankung, die durch das Bakterium *T. whipplei* verursacht wird [4]. Histologisch ist der Morbus Whipple klassischerweise durch PAS-positive Einschlüsse in Makrophagen der duodenalen Lamina propria^2^ gekennzeichnet [2]. Erstmals beschrieben wurde die Erkrankung im Jahr 1907 von dem US-amerikanischen Pathologen und Nobelpreisträger^3^ George Hoyt Whipple [5]. Interessanterweise gelang die Identifikation des Erregers erst 1992 [4], ca. 85 Jahre nach der Erstbeschreibung, die Kultivierung erfolgte wiederum erst im Jahr 2000 [6].

Leitbefunde des Morbus Whipple sind Arthritiden, Diarrhoen und Gewichtsverlust [2, 3]. Zu Krankheitsbeginn stehen vor allem Gelenkmanifestationen (Prodromalstadium) im Vordergrund, bevor gastrointestinale Symptome typischerweise erst Jahre (6–7 Jahre) später auftreten [1, 7]. Im weiteren Verlauf kann eine chronische Malabsorption zur Kachexie führen. Die Gelenke zeigen eine Oligo- oder Polyarthritis mit migratorischem Befall und episodenhaftem Charakter (akuter Beginn, etwa 1 Woche anhaltend) [7, 8]. Vorwiegend sind große Gelenke (v. a. Knie- und Handgelenke) betroffen, während ein Befall kleiner Gelenke (insbesondere Zehengelenke^4^) eher für eine primär rheumatologische Grunderkrankung spricht [7, 8]. In der Regel verlaufen die Arthritiden nicht erosiv, obwohl auch derartige Fälle beschrieben sind [7, 9]. Darüber hinaus sind auch axiale Befallsmuster im Sinne einer SpA, Sakroiliitis oder Spondylodiszitis berichtet worden [2, 7, 10, 11]. Zusätzlich können weitere unspezifische Symptome wie Bauchschmerzen, Fieber, Lymphadenopathie und Anämie (chronische Entzündung, Malabsorption) bestehen [3]. Auch die Entwicklung einer Splenomegalie ist beschrieben [3]. Interessanterweise betrifft die Erkrankung meistens Männer im mittleren und höheren Lebensalter [2, 3]. Angesichts der Seltenheit des Morbus Whipple beträgt die mittlere Dauer bis zur Diagnosestellung etwa 7 Jahre [7]. Das gleichzeitige Auftreten von Diarrhoen oder Gewichtsverlust verkürzt das diagnostische Intervall in der Regel (siehe Fall 4) [3].

Eine wichtige Krankheitsmanifestation ist die Infektion des zentralen Nervensystems (ZNS) durch *T. whipplei*, die ein breites klinisches Spektrum aufweisen (z. B. kognitive Störungen, Kopfschmerzen, Depression) und in bis zu 50 % der Fälle nachgewiesen werden kann [3, 12]. Es bestehen häufig okkulte ZNS-Infektionen, weshalb bei Diagnosestellung eine Lumbalpunktion mit PCR-Untersuchung des Liquors empfohlen wird [2, 13]. Als pathognomonisch für eine ZNS-Infektion gelten okulomastikatorische Myorhythmien (okuläre Vergenzbewegungen mit gleichzeitiger Kontraktion der Kaumuskulatur) [12, 14].

Zusätzlich kann *T. whipplei* auch kardiale Strukturen befallen und gilt als häufigster Erreger einer isolierten Blutkultur-negativen (!) Endokarditis [3, 5]. Die Sicherung der Diagnose gelingt dann durch eine PCR und histopathologische Untersuchung der operierten Klappen mittels PAS-Reaktion und Immunhistochemie [15–17].

In Abgrenzung zur systemischen Verlaufsform des Morbus Whipple sind auch diverse lokalisierte Infektionen durch *T. whipplei* beschrieben (z. B. Enzephalitis, Uveitis) [2]. Anamnestische und klinische Hinweise einer gastrointestinalen Beteiligung fehlen in diesen Fällen und die histologische Untersuchung von Duodenalbiopsaten ist unauffällig. Die Diagnose basiert dann auf der Untersuchung anderer Gewebe oder steriler Flüssigkeiten, z. B. durch den molekularbiologischen Nachweis von *T. whipplei* mittels PCR (z. B. Gelenkpunktat) bzw. Histologie mit PAS-Reaktion (z. B. Lymphknoten) [3]. Aus rheumatologischer Sicht sind hauptsächlich isolierte Gelenkinfektionen (Arthritis) und Spondylodiszitiden bedeutsam [2]. Übergänge von lokalisierten Infektionen zur systemischen Verlaufsform (Morbus Whipple) mit gastrointestinaler Beteiligung sind beschrieben worden [15].

Chronische Infektionen durch *T. whipplei* mit Gelenkbefall werden häufig als primär entzündlich-rheumatische Systemerkrankung (z. B. RA oder PsA) fehlgedeutet und zunächst immunsuppressiv therapiert. Gerade der refraktäre und progrediente Verlauf „trotz“ Einsatz verschiedener Basismedikamente bei Patient:innen (insbesondere bei männlichem Geschlecht) mit „seronegativer RA“ ist jedoch verdächtig! Hier sollte die Differentialdiagnose einer Infektion mit *T. whipplei* auch bei fehlender gastrointestinaler Symptomatik nicht frühzeitig verworfen werden. Stattdessen sollte in diesen Fällen gezielt nach weiteren Krankheitsmanifestationen eines Morbus Whipple (z. B. ZNS-Beteiligung) gefahndet werden. Häufig führt das fehlende Therapieansprechen unter Annahme einer unzureichenden Immunsuppression jedoch zu einer Therapieintensivierung. Eine Verbesserung des klinischen Zustandes bleibt dennoch aus. Diesen Kreislauf gilt es zu durchbrechen, indem „red flags“ als Hinweis auf eine *T.-whipplei*-Infektion bei der klinischen Evaluation berücksichtigt werden (Tab. 2).

**Table 2:** 

Chronische seronegative Polyarthritis ohne Ansprechen auf immunsuppressive Therapien (insbesondere bei männlichen Patienten ohne Befall der kleinen Gelenke und persistierender Inflammation sowie Fehlen von Antikörpern gegen zyklische citrullinierte Peptide)
Migratorische Polyarthritis (seltener Oligoarthritis) der großen Gelenke unklarer Genese mit akuten intermittierenden Episoden (ca. 1 Woche anhaltend)
Arthritis mit Gewichtsverlust und chronischen Diarrhoen
Demenz mit supranukleärer Ophthalmoplegie (v. a. vertikal) und Myoklonien
Okulomastikatorische Myorhythmie oder okulofaziale skelettale Myorhythmie
Blutkultur-negative Endokarditis

### Diagnostik des Morbus Whipple und lokalisierter Infektionen

Die aktuell in Überarbeitung befindliche deutsche S2k-Leitlinie^5^ „Gastrointestinale Infektionen und Morbus Whipple“ aus 2015 empfiehlt als erste diagnostische Maßnahme bei Verdacht auf Morbus Whipple eine ÖGD mit histologischer Untersuchung von ≥ 5 Duodenalbiopsaten mittels PAS-Reaktion [13]. Zur Bestätigung kann eine PCR-Untersuchung der Duodenalbiopsate (auch aus differentialdiagnostischen Gründen^6^) ergänzt werden [13]. Es ist zu betonen, dass eine positive PCR-Untersuchung von Duodenalbiopsaten in Abwesenheit von typischen histologischen Veränderungen die Diagnose eines Morbus Whipple nicht beweist, sondern auch eine reine Kolonisation mit dem Erreger anzeigen kann [3, 13]. Auf der anderen Seite schließt eine normale Duodenalhistologie eine chronische *T.-whipplei*-Infektion nicht aus (wie die beschriebenen Fälle demonstrieren) [2, 11]. Bei anhaltendem Verdacht sollte bei „chronischer seronegativer Polyarthritis“ eine wiederholte diagnostische Gelenkpunktion betroffener Gelenke mit PCR-Untersuchung auf *T. whipplei* angestrebt werden [11, 13]. Bei Diagnosestellung wird zusätzlich eine Liquoruntersuchung mittels PCR zum Ausschluss einer okkulten ZNS-Infektion in allen Fällen empfohlen [3, 13]. Untersuchungen von Speichel und Stuhl mittels PCR werden in der Leitlinie aufgrund der möglichen apathogenen Kolonisation nicht empfohlen.

Tison et al. [11] verzichten im Gegensatz zur deutschen Leitlinie auf die ÖGD und schlagen bei entsprechendem Verdacht und Nachweis von Gelenkergüssen bereits eine initiale Gelenkpunktion mit PCR-Untersuchung („localized Whipple’s arthritis“) vor. Ergänzend empfehlen die Autor:innen als Screeningmethode auch die PCR aus Stuhl und Speichel und befürworten die ÖGD nur bei Hinweisen für systemische bzw. gastrointestinale Manifestationen (Abklärung „classic Whipple’s disease“).

Lagier und Raoult [2] fügen als weiteren diagnostischen Standard die PCR aus dem Urin hinzu. Als mögliche bildgebende Diagnostik wird die Fluordesoxyglukose-Positronenemissionstomographie(^18^F‑FDG-PET)-CT-Untersuchung zur Darstellung von duodenaler Beteiligung, mesenterialer Lymphadenopathie, Endokarditis und Hypometabolismus bei ZNS-Beteiligung vorgeschlagen.

Anhand der präsentierten Fälle wird ersichtlich, dass eine *T.-whipplei*-Infektion nicht nur einen oligoarthritischen Befall großer Gelenke, sondern eben auch eine symmetrische Polyarthritis kleiner Gelenke mit teils erosivem Verlauf verursachen kann. Unter Umständen erfüllen diese Patient:innen dann formal die ACR-/EULAR-Klassifikationskriterien für die RA.

Vor diesem Hintergrund kann aus Sicht der Autor:innen eine diagnostische Gelenkpunktion inklusive Whipple-PCR bei Oligoarthritis und Anti-CCP-negativer Polyarthritis mit fehlendem Ansprechen auf drei Basismedikamente (davon mindestens ein bDMARD oder tsDMARD) erwogen werden.

### Therapie des Morbus Whipple und lokalisierter Infektionen

In der deutschen Leitlinie zur Therapie des Morbus Whipple wird initial eine intravenöse Therapie mit Ceftriaxon 2 g täglich über 14 Tage und anschließender oraler Therapie mit Cotrimoxazol 960 mg 2 × täglich für 1 Jahr empfohlen [13]. Eine gesonderte Empfehlung für die Therapie der isolierten Arthritis durch *T. whipplei* wird nicht beschrieben [13].

Lagier und Raoult^7^ [2] empfehlen in ihrem Review aus 2018 hingegen eine orale Kombinationstherapie mit Doxycyclin 200 mg täglich und Hydroxychloroquin 600 mg täglich für 1 Jahr. Anschließend wird von den Autoren eine lebenslange Erhaltungstherapie mit Doxycyclin vorgeschlagen. Die lokalisierte Endokarditis und ZNS-Infektion (Enzephalitis) sollen aufgrund des Rezidivrisikos analog zum Morbus Whipple ebenfalls lebenslang therapiert werden. Für den Einsatz von Cotrimoxazol wird keine Indikation gesehen, da dem Trimethoprim das entsprechende molekulare Target bei *T. whipplei* fehle und eine Resistenzentwicklung in mehreren Fällen beschrieben wurde [2]. Im Falle einer lokalisierten Arthritis wird eine orale Kombinationstherapie mit Doxycyclin 200 mg täglich und Hydroxychloroquin 600 mg täglich für 12 bis 18 Monate empfohlen [2].

Klinisch ist unter der antiinfektiven Therapie kurzfristig (Stunden) auf Jarisch-Herxheimer-Reaktionen zu achten, wie wir es bei einem Patienten (Fall 4) beobachtet haben. Mittelfristig (Wochen) kann ein entzündliches Immunrekonstitutionssyndrom (IRIS) mit lokalisierter (z. B. Arthritis) und systemischer Entzündungsreaktion (z. B. Fieber) auftreten [13]. Im Falle solcher Symptomatik sollte die Behandlung aufgrund des potenziell tödlichen Verlaufs in einem spezialisierten Zentrum erfolgen.

Die deutsche Leitlinie empfiehlt bei intestinalem Befall eine endoskopisch-bioptische Verlaufskontrolle nach 6 und 12 Monaten antibiotischer Therapie [13]. Die *T.-whipplei*-Arthritis soll klinisch und laborchemisch kontrolliert werden [13]. Bei initial positivem Liquorbefund sollte nach 12 Monaten eine Verlaufskontrolle durch eine erneute PCR-Untersuchung des Liquors erfolgen [13].

Beim Morbus Whipple empfehlen Lagier und Raoult hingegen zur Kontrolle (bei fehlenden klinischen Zeichen) die Untersuchung von Stuhl, Speichel und Urin zweimal pro Jahr [2, 18]. Für die isolierte Arthritis werden lediglich klinische „Verlaufskontrollen“ empfohlen, um einen möglichen Übergang in einen Morbus Whipple mit Indikation zur lebenslangen antibiotischen Therapie zu detektieren [2]. Möglicherweise kann eine immunsuppressive Therapie für den Übergang eine Rolle spielen [11]. Bei der Verlaufskontrolle von ZNS-Infektionen könnte auch die ^18^F‑FDG-PET-CT von Bedeutung sein [15].

## Fazit für die Praxis

- Therapierefraktäre Polyarthritiden können auf eine chronische Infektion mit *T. whipplei *hindeuten. Nach weiteren anamnestischen (z. B. Gewichtsverlust) und klinischen (z. B. Kachexie) Hinweisen auf einen Morbus Whipple sollte gesucht werden.
- Insbesondere das fehlende Ansprechen auf verschiedene Immunsuppressiva bei Patient:innen mit „seronegativer rheumatoider Arthritis“ ist verdächtig.
- Wir empfehlen eine diagnostische Gelenkpunktion mit PCR-Untersuchung des Gelenkpunktates auf *T. whipplei* bei therapierefraktärer Oligoarthritis oder Anti-CCP-negativer Polyarthritis (mindestens drei DMARDs, darunter ein bDMARD oder tsDMARD).

## Footnotes

1 Periodsäure-Schiff-Reaktion („periodic acid Schiff reaction“), umgangssprachlich auch als „PAS-Färbung“ bezeichnet.

2 Hierbei handelt es sich um den klassischen Befund eines Morbus Whipple. Es ist zu betonen, dass PAS-positive Einschlüsse in Makrophagen auch in anderen Geweben (z. B. Lymphknoten) zu finden sind und so zur Diagnose führen können.

3 Den Nobelpreis für Physiologie oder Medizin erhielten G. H. Whipple, G. R. Minot und W. P. Murphy im Jahr 1934 für ihre Beiträge zur Erforschung der perniziösen Anämie.

4 Eine aktuelle Arbeit von Feurle et al. [8] untersuchte den Wert rheumatologischer Symptome und Befunde bei der differentialdiagnostischen Abgrenzung des Morbus Whipple gegenüber entzündlich-rheumatischer Systemerkrankungen wie der rheumatoiden Arthritis.

5 Die Gültigkeit der deutschen S2k-Leitlinie „Gastrointestinale Infektionen und Morbus Whipple“ endete am 30.01.2020. Die Fertigstellung der neuen Leitlinie ist für den 31.07.2023 geplant.

6 Hierbei sind v. a. atypische Mykobakteriosen zu berücksichtigen [3].

7 Der Arbeitsgruppe um D. Raoult gelang im Jahr 2000 die erste Kultivierung von *T. whipplei *[6].

## References

[1] Puechal X (2001). Whipple disease and arthritis. Curr Opin Rheumatol.

[2] Lagier JC, Raoult D (2018). Whipple’s disease and Tropheryma whipplei infections: when to suspect them and how to diagnose and treat them. Curr Opin Infect Dis.

[3] Marth T, Moos V, Muller C, Biagi F, Schneider T (2016). Tropheryma whipplei infection and Whipple’s disease. Lancet Infect Dis.

[4] Relman DA, Schmidt TM, MacDermott RP, Falkow S (1992). Identification of the uncultured bacillus of Whipple’s disease. N Engl J Med.

[5] Fenollar F, Puechal X, Raoult D (2007). Whipple’s disease. N Engl J Med.

[6] Raoult D, Birg ML, La Scola B, Fournier PE, Enea M, Lepidi H, Roux V, Piette JC, Vandenesch F, Vital-Durand D, Marrie TJ (2000). Cultivation of the bacillus of Whipple’s disease. N Engl J Med.

[7] Puechal X (2016). Whipple’s arthritis. Joint Bone Spine.

[8] Feurle GE, Moos V, Stroux A, Gehrmann-Sommer N, Poddubnyy D, Fiehn C, Schneider T (2021). Differential diagnostic value of rheumatic symptoms in patients with Whipple’s disease. Sci Rep.

[9] Krusche M, Boro D, Bertolini J, Kötter I (2019). Seltenes erosives Arthritis- und Dermatitissyndrom bei Morbus Whipple. Z Rheumatol.

[10] Desmurs M, Petit H, Gottenberg J-E (2019). SAT0453 joint manifestations of Whipple’S disease: clinical and radiological presentation of 19 patients. Ann Rheum Dis.

[11] Tison A, Preuss P, Leleu C, Robin F, Le Pluart A, Vix J, Le Mélédo G, Goupille P, Gervais E, Cormier G, Albert J-D, Perdriger A, Bouvard B, Berthelot J-M, Foulquier N, Saraux A (2021). Rheumatological features of Whipple disease. Sci Rep.

[12] El-Abassi R, Soliman MY, Williams F, England JD (2017). Whipple’s disease. J Neurol Sci.

[13] Hagel S, Epple HJ, Feurle GE, Kern WV, Lynen Jansen P, Malfertheiner P, Marth T, Meyer E, Mielke M, Moos V, von Muller L, Nattermann J, Nothacker M, Pox C, Reisinger E, Salzberger B, Salzer HJ, Weber M, Weinke T, Suerbaum S, Lohse AW, Stallmach A, Weitere Mitglieder der Leitlinienkommission sind am Ende des Beitrags g (2015). S2k-Leitlinie Gastrointestinale Infektionen und Morbus Whipple. Z Gastroenterol.

[14] Bally JF, Meneret A, Roze E, Anderson M, Grabli D, Lang AE (2018). Systematic review of movement disorders and oculomotor abnormalities in Whipple’s disease. Mov Disord.

[15] Lagier JC, Cammilleri S, Raoult D (2016). Classic Whipple’s disease diagnosed by (18)F-fluorodeoxyglucose PET. Lancet Infect Dis.

[16] Celard M, de Gevigney G, Mosnier S, Buttard P, Benito Y, Etienne J, Vandenesch F (1999). Polymerase chain reaction analysis for diagnosis of Tropheryma whippelii infective endocarditis in two patients with no previous evidence of Whipple’s disease. Clin Infect Dis.

[17] Fenollar F, Celard M, Lagier JC, Lepidi H, Fournier PE, Raoult D (2013). Tropheryma whipplei endocarditis. Emerg Infect Dis.

[18] Moter A, Janneck M, Wolters M, Iking-Konert C, Wiessner A, Loddenkemper C, Hartleben B, Lutgehetmann M, Schmidt J, Langbehn U, Janssen S, Geelhaar-Karsch A, Schneider T, Moos V, Rohde H, Kikhney J, Wiech T (2019). Potential role for urine polymerase chain reaction in the diagnosis of Whipple’s disease. Clin Infect Dis.

